# A Bacterial Epigenetic Switch in Non-typeable *Haemophilus influenzae* Modifies Host Immune Response During Otitis Media

**DOI:** 10.3389/fcimb.2020.512743

**Published:** 2020-10-23

**Authors:** Frank H. Robledo-Avila, Juan de Dios Ruiz-Rosado, Santiago Partida-Sanchez, Kenneth L. Brockman

**Affiliations:** ^1^Center for Microbial Pathogenesis, Abigail Wexner Research Institute at Nationwide Children's Hospital, Columbus, OH, United States; ^2^Department of Biochemistry and Immunology, National Technological Institute of Oaxaca, Oaxaca, Mexico; ^3^Department of Pediatrics, College of Medicine, The Ohio State University, Columbus, OH, United States; ^4^Department of Microbiology and Immunology, Medical College of Wisconsin, Milwaukee, WI, United States

**Keywords:** NTHI, otitis media (OM), phasevarion, chinchilla, neutrophil, biofilm

## Abstract

Non-typeable *Haemophilus influenzae* (NTHi) causes multiple diseases of the human airway and is a predominant bacterial pathogen of acute otitis media and otitis media in which treatment fails. NTHi utilizes a system of phase variable epigenetic regulation, termed the phasevarion, to facilitate adaptation and survival within multiple sites of the human host. The NTHi phasevarion influences numerous disease-relevant phenotypes such as biofilm formation, antibiotic resistance, and opsonization. We have previously identified an advantageous selection for a specific phasevarion status, which significantly affects severity and chronicity of experimental otitis media. In this study, we utilized pure cultures of NTHi variants in which *modA* was either locked ON or locked OFF, and thus *modA* was unable to phase vary. These locked variants were used to assess the progression of experimental otitis media and define the specific immune response induced by each subpopulation. Although the initial disease caused by each subpopulation was similar, the immune response elicited by each subpopulation was unique. The *modA2* OFF variant induced significantly greater activation of macrophages both *in vitro* and within the middle ear during disease. In contrast, the *modA2* ON variant induced a greater neutrophil extracellular trap response, which led to greater killing of the *modA2* ON variant. These data suggest that not only does the NTHi phasevarion facilitate adaptation, but also allows the bacteria to alter immune responses during disease. Understanding these complex bacterial-host interactions and the regulation of bacterial factors responsible is critical to the development of better diagnostic, treatment, and preventative strategies for these bacterial pathogens.

## Introduction

Human associated bacteria must be able to endure a wide range of stresses in order to survive and persist within their human host. Stressors include changes in the microenvironment, such as temperature, pH, and nutrient availability, as well as host humoral and innate immune responses. Bacteria that reside within the airways experience additional stressors which include ciliary clearance, changes in humidity or gas concentrations, and the production of mucus. Bacteria have developed a range of tactics to adapt to and overcome these stressors. Furthermore, several mucosal associated human pathogens have developed a mechanism of bacterial epigenetic regulation, termed the phasevarion, which facilitates rapid, coordinated adaptation to microenvironmental fluctuations and host defenses (Srikhanta et al., 2005).

The phasevarion, or phase variable regulon, has been identified in several diverse mucosal-associated bacteria, which include *Haemophilus influenzae, Neisseria species, Moraxella catarrhalis, Streptococcus pneumoniae, Helicobacter pylori*, and *Kingella kingae* (Srikhanta et al., 2005, 2009, 2011, 2017a; Blakeway et al., 2014; Manso et al., 2014). Regulation of the bacterial phasevarion involves a phase variable DNA methyltransferase, Mod, which when active alters the expression of a large set of genes. Due to phase variation, expression of the functional Mod protein can randomly turn on and off, or switch, which results in two phenotypically distinct bacterial subpopulations within the bacterial population. These two subpopulations are referred to as ON and OFF, for review see (Srikhanta et al., 2010; Tan et al., 2016; Atack et al., 2018). Recent work has shown that each of these distinct subpopulations differ in the expression of known virulence factors and responses to disease-related stresses *in vitro* (Atack et al., 2015a, 2019a; Brockman et al., 2017, 2018; Srikhanta et al., 2017b). Phasevarion-mediated changes in gene expression not only impact bacterial physiology, but also present an important challenge for vaccine development. Regulation of potential bacterial antigens by the phasevarion mechanism could have significant implications on the selection and efficacy of vaccine targets. In addition, the phasevarions of *H. pylori* and *S. pneuominae* have been shown to affect persistence and virulence in murine models of disease (Gauntlett et al., 2014; Manso et al., 2014). The work presented herein seeks to further define the role of the ModA2 phasevarion of non-typeable *Haemophilus influenzae* on innate immune response during experimental otitis media.

Non-typeable *Haemophilus influenzae* (NTHi) asymptomatically colonizes the nasopharynx of most of the human population, but can also cause multiple respiratory tract infections, which include otitis media, rhinosinusitis, conjunctivitis, bronchitis, and exacerbations in patients with chronic obstructive pulmonary disease (COPD) or cystic fibrosis (CF) (Ruohola et al., 2006; Murphy et al., 2009). Although less common, some NTHi can also cause invasive disease (Van Eldere et al., 2014). NTHi is a predominant pathogen of acute otitis media and cause of recurrent otitis media and otitis media in which treatment fails (Haggard, 2008; Leibovitz, 2008; Welp and Bomberger, 2020). Otitis media occurs when bacteria within the nasopharynx are able to ascend the Eustachian tube and gain access to the middle ear space, often due to the presence of predisposing factors, such as concurrent viral infection or young age (Harmes et al., 2013). No vaccine is currently available for the treatment or prevention of infections due to NTHi, however, global efforts are underway to identify effective vaccine targets. Antibiotics are the current first-line treatment for NTHi infections but are often ineffective due to formation of antibiotic resistant bacterial biofilms (Slinger et al., 2006; Swords, 2012). Furthermore, widespread use of antibiotics to treat NTHi infections, is of great concern due to the rapid rise in antibiotic and multi-drug resistant bacteria (Murphy, 2015; Bakaletz and Novotny, 2018). As such, there is a strong need for improved or alternative treatment modalities for these diseases.

NTHi utilize a wide variety of mechanisms to evade host immunity and persist within their host. Resistance to oxidative stress increases *in vitro* survival from neutrophil killing and promotes persistence in animal models of disease (Hong et al., 2009; Juneau et al., 2015; Brockman et al., 2017). Biofilm formation results in increased antibiotic resistance of numerous NTHi strains and protects bacteria from a range of exogenous stressors. NTHi also produce multiple surface components, such as outer membrane proteins (OMPs) and lipooligosaccharides (LOS). OMPs facilitate bacterial adherence to mucosal surfaces during both colonization and infection, and can induce innate immune response (Berenson et al., 2005; Punturieri et al., 2006). LOS has been shown to be both immunostimulatory as well as immunoevasive, dependent on the composition and structure of the molecule (Hong et al., 2009; Juneau et al., 2011; Jackson et al., 2019; Ng et al., 2019). Regulation of the genes involved in each of these processes can have substantial impact on pathogenesis of this human pathobiont.

Initial studies into the phasevarion of NTHi found that within multiple diverse collections of clinical isolates, up to two-thirds of NTHi had an active phasevarion (Atack et al., 2015a). Since then, NTHi phasevarions were shown to regulate numerous disease-related processes, which include biofilm formation, resistance to oxidative stress, opsonization and antibiotic sensitivity (Atack et al., 2015a; VanWagoner et al., 2016; Brockman et al., 2017, 2018). The NTHi phasevarion has also been studied *in vivo*, where NTHi strain 723 was shown to regulate pathogenesis and disease severity in a chinchilla model of experimental otitis media (Atack et al., 2015a; Brockman et al., 2016). In these early studies, we found that NTHi shift to a preferred *modA2* ON status within the chinchilla middle ear fluids and that this shift in overall phasevarion status (from OFF to ON) resulted in significantly greater disease severity within the middle ear compared to when there was no change in the overall population status. While the specific factors that result in this increased severity remain unknown, we propose that there may be a unique host immune response mounted against each discrete *modA* subpopulation. To test this hypothesis, herein we investigate how the two different *modA2* subpopulations interface with the host and affect innate immune response using the chinchilla model of experimental otitis media. While all previous studies with this model used phase variable populations, which were able to switch *modA2* status, in these studies we now use locked variants that are unable to phase vary or switch status. Our findings in the chinchilla are further compared with human innate immune cells.

## Results

### NTHi Strain 723 Locked Variants Induce Otitis Media

**Figure 1:**
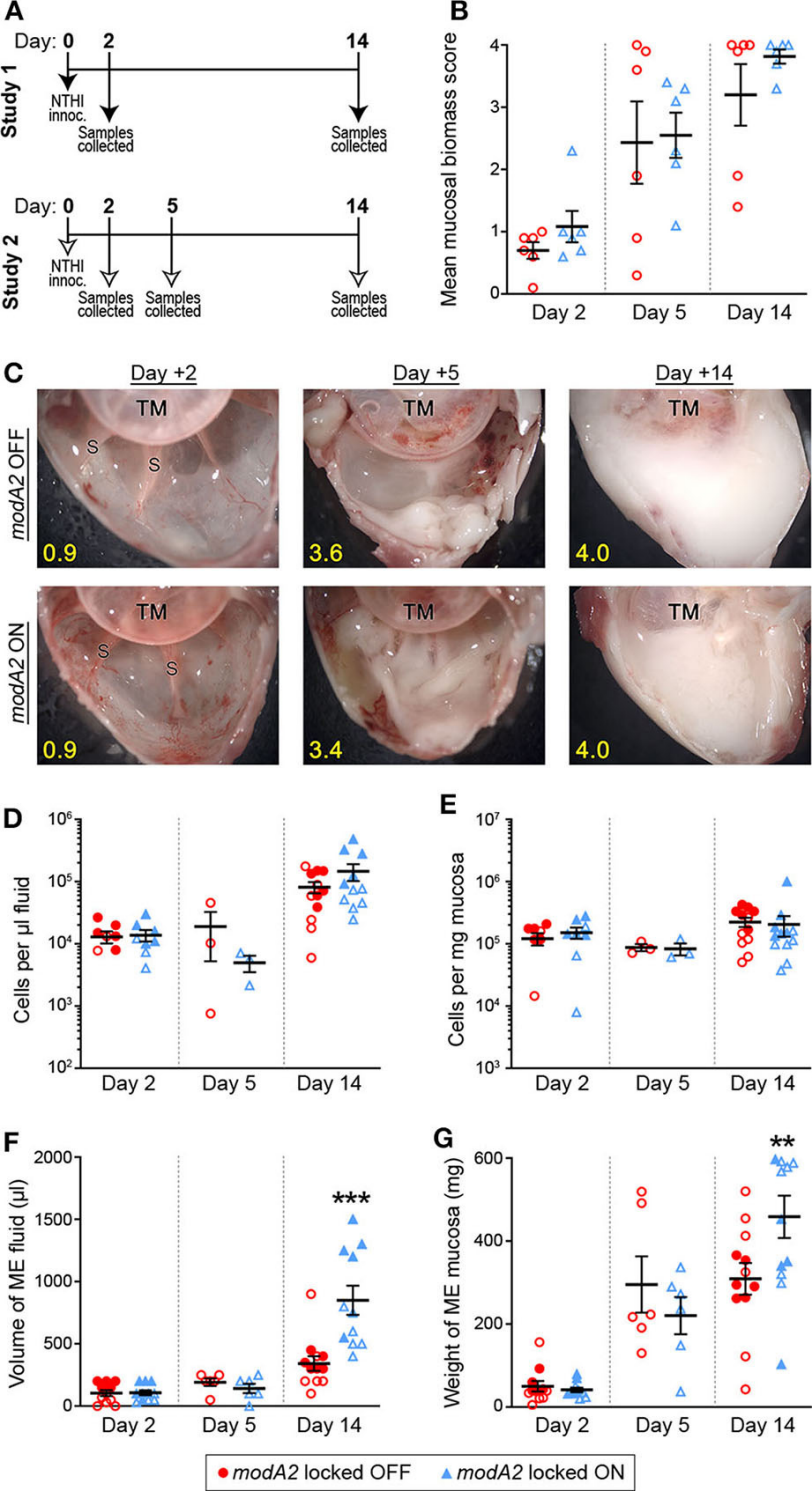
Chinchilla model of otitis media with NTHi strain 723 *modA2* locked variants. **(A)** Chinchillas were challenged via transbullar inoculation of ~750 CFU NTHi directly into the middle ear space. Samples were collected post-mortem at days 2, 5, or 14 after challenge. **(B)** At time of sample collection, middle ears were opened and imaged to assess gross mucosal biomass. Biomass was blindly scored on a scale of 0 (no biomass) to 4 (filled with biomass). Biomass increased up to a peak biomass at 14 days post challenge. No significant differences were observed between the variants. **(C)** Representative images of middle ears at 2, 5, and 14 days after challenge with *modA2* locked variants. A buildup of biofilm biomass is indicated by the solid white material composed of bacterial and host cells. Average score for each representative image indicated in yellow. TM, tympanic membrane; S, boney septa. **(D,E)** Density of host cells within the middle ear fluid **(D)** or mucosa **(E)**. There were no significant differences between the variants at any of the days assessed. **(F)** Volume of fluid collected from the middle ear. There was no significant difference in the amount of fluid at day 2 or day 5 after challenge for either variant. At day 14, ears challenged with *modA2* ON had significantly more fluid than those challenge with *modA2* OFF. **(G)** Weight of solid biomass collected from the middle ear. There was an increase in mucosal weight over the course of disease, which correlated with mucosal biomass scores. Ears that received *modA2* ON had significantly more mucosal biomass, by weight, at Day 14 compared to those challenged with *modA2* OFF. **p < 0.05, ***p < 0.001, unpaired *t*-test.

To better define the individual contribution of each variant (*modA2* OFF vs. *modA2* ON) to disease pathology, this work assessed disease induced by genetically modified variants of NTHi that are unable to phase vary, or switch status (Brockman et al., 2018). Disease severity was evaluated using an established chinchilla model of experimental otitis media (Bakaletz, 2009). Chinchillas were challenged with pure NTHi strain 723 populations that were either *modA2* locked OFF or *modA2* locked ON, and thus *modA2* was unable to phase vary. In two independent studies, cohorts of 9 adult chinchillas each were challenged transbullarly with the *modA2* OFF or *modA2* ON population (750 ± 50 CFU NTHi per ear) and blindly assessed for signs of disease daily. Inoculums were confirmed by serial dilution and plating, and there was no significant difference in the number of bacteria delivered to each cohort at time of challenge. All animals had bilateral middle ear effusions by 24 h after challenge, as expected following direct inoculation of NTHi into the middle ear. Animals were euthanized 2 days after challenge, to assess early stages of disease, 5 days after challenge, to assess a developing disease, or 14 days after challenge near the peak of infection and assessed for overall signs of experimental disease (Figure 1A).

Gross middle ear mucosal biomass was imaged and blindly scored on a scale of 0 to 4 for relative biomass, in which a score of 0 indicated no biofilm was present and a score of 4 indicated that >75% of the middle ear space was filled with biofilm (Goodman et al., 2011). There was no significant difference in mean mucosal biomass scores between the ears that received the *modA2* OFF variant compared to those that received the *modA2* ON variant, at any of the days assessed (Figure 1B). However, there was a consistent increase in mucosal biofilm burden from 2 to 5 days post challenge and 5 to 14 days post challenge, irrespective of *modA2* status. These results were similar to biofilm formation reported during experimental otitis media induced by other NTHi strains (Hong et al., 2009, 2019; Novotny et al., 2017). Representative images of middle ears challenged with the *modA2* OFF or *modA2* ON variants are shown in Figure 1C. At day 14 post-infection, white mucosal biofilm biomass, composed of bacterial and host immune cells, completely occluded the mucosal surface and boney septa that were visible at day 2. These results were consistent with previous studies in which phase variable populations that did not shift status resulted in equally similar disease severity, albeit significantly milder than the experimental disease that occurs when the *modA2* status of the population shifts within the middle ear (Brockman et al., 2016).

Middle ear fluids (MEF) and middle ear mucosa with any adherent biomass (MEM) were collected and the viable host cells within each sample were enumerated. There was no difference in the cell density or total amount of sample collected between the cohorts at 2 days after challenge (Figures 1D–G). The density of cells in the middle ear fluids increased from day 2 to day 14 after challenge; however, there was no difference in the cell density between challenge cohorts (Figures 1D,E). Fourteen days after challenge, animals that received the *modA2* ON variant had significantly more middle ear fluid and significantly greater mucosal mass compared to animals that received the *modA2* OFF variant (Figures 1F,G). Together these results suggested that the overall burden within the middle ear at day 14 was greater in ears challenged with *modA2* ON, yet the specific host cells, immune or epithelial, that contributed to this observation were unclear. Additionally, viable bacteria within the middle ear 14 days after challenge were enumerated. There was no significant difference in the relative bacterial load within the middle ear fluids (CFU/uL) or middle ear mucosal samples (CFU/mg) of animals challenged with *modA2* locked OFF compared to *modA2* locked ON (Supplementary Figure 1). These results suggested that differences in disease severity or host response to each of these variants was not due to differences in relative bacterial abundance, but rather due to changes associated with *modA2* status.

### Infiltration of Immune Phagocytes Into the Middle Ear

**Figure 2:**
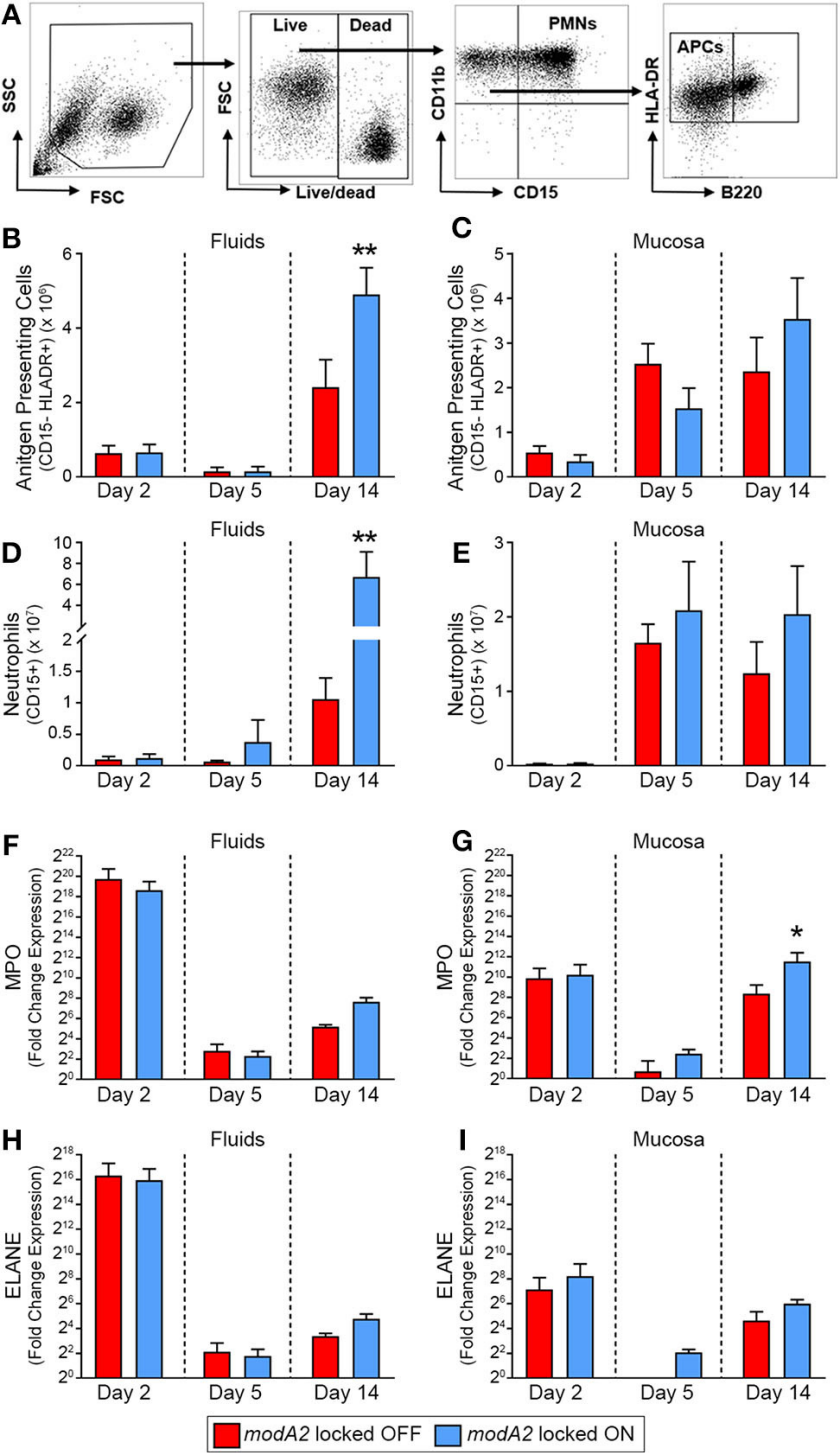
Immune cell populations within the middle ear over the course of experimental otitis media. **(A)** Representative dot plots of the gating strategy used to quantify neutrophils (CD11b+ CD15+) and antigen presenting cells (CD11b+ CD15– B220– HLADR+). **(B–E)** Total number of immune cells within the middle ear. Antigen presenting cells (APCs) in the middle ear fluids **(B)** and mucosal biomass **(C)** and neutrophils within the middle ear fluids **(D)** and mucosal biomass **(E)**. Animals challenged with *modA2* ON had significantly more APCs and neutrophils within the middle ear fluids at Day 14, compared to those challenged with *modA2* OFF. **(F–I)** Fold change in gene expression relative to naïve middle ear mucosa. Expression of myeloperoxidase, MPO, in the middle ear fluids **(F)** and mucosal biomass **(G)**, and expression of neutrophil elastase, ELANE, in the middle ear fluids **(H)** and mucosal biomass **(I)**. *n* = 6 ears per study. *p < 0.1, **p < 0.05, unpaired *t*-test.

To next investigate the recruitment of immune cells into the middle ear over the course of disease, we determined the number of live CD11b+ CD15– HLADR+ antigen presenting cells (APCs) and live CD11b+ CD15+ granulocytes (neutrophils) in the samples collected from each animal study (Figure 2A). During the course of disease, the microenvironment and composition of the middle ear fluids and mucosal biofilm are very different and can significantly impact the phenotypes of the bacteria and immune cells present. As such, the fluids and mucosal samples were analyzed independently. When comparing ears that received the *modA2* OFF vs. *modA2* ON variants, there was no difference in the number of APCs in either the middle ear fluids or mucosal samples 2 days post-challenge (Figures 2B,C). Five days after challenge there was again no difference in the number of APC between cohorts. Interestingly, there was an overall marked increase in APCs within the mucosal biomass from day 2 to day 5 post-challenge, which did not occur within the middle ear fluids. Fourteen days after bacterial challenge significantly more APCs were present within the middle ear fluids of animals challenged with *modA2* ON compared to *modA2* OFF (*p* = 0.026, *t*-test; Figure 2B). A similar trend was observed with the mucosal samples; however, the difference was not statistically significant at this time point (Figure 2C). Comparable results were observed with CD15+ neutrophils. There were no significant differences in total neutrophil cell counts in either sample location (MEF vs. MEM) at 2 or 5 days post infection. However, 14 days after challenge, middle ear fluids from animals challenged with the *modA2* ON variant had significantly more CD15+ granulocytes compared to those challenged with the *modA2* OFF variant (*p* = 0.024, *t*-test; Figure 2D). A similar trend was observed in the mucosal samples; however, the results were not statistically significant (Figure 2E). The overall patterns of myeloid cell abundance correlated with total host cells present in the samples, and further suggested that CD15+ granulocytes, and to a lesser extent APCs, are the primary source of infiltrating immune cells into the middle ear at this time point.

### Immune Cell Gene Expression

To further assess the composition and activation state of cells retrieved from the middle ear, RNA was isolated from a fraction of the cells collected as part of the second study. Gene expression was assessed for cells in both the middle ear fluids and mucosal samples collected 2, 5 or 14 days after challenge with the *modA2* OFF or *modA2* ON variants. Expression of pro-inflammatory cytokines (IL1A, IL1B, IL6, CXCL8, IL23, & TNF) was determined for the bulk (mixed) cell populations collected from the middle ear fluids and mucosal biomass samples, with each sample assessed separately. There were no significant differences between cohorts in the expression of pro-inflammatory cytokines at the time points assessed (Supplementary Figure 2). However, all samples from animals that received either *modA2* variant had significantly greater expression of all inflammatory genes assessed, compared to middle ear mucosa from unchallenged naïve animals (Supplementary Figure 2). One of the animals euthanized at day 2 appeared to have a substantially increased response across all cytokines, which may be due to the heterogeneous nature of this outbred, mixed-sex animal model. We next evaluated relative gene expression of the genes that encode myeloperoxidase and neutrophil elastase, MPO and ELANE, respectively. Both myeloperoxidase and neutrophil elastase are located in primary (azurophil) granules and have important roles in bacterial killing and release of neutrophil extracellular traps (NETs). A massive and rapid increase in the expression of both MPO and ELANE was observed in middle ear fluids and mucosa harvested 2 days after challenge, compared to naïve mucosa (Figures 2F–I). Both *modA2* variants produced this initial response to a comparable degree. In the middle ear fluids, there was a rapid decrease in relative expression of MPO and ELANE from day 2 to day 5 post-challenge and a moderate increase in relative expression from day 5 to day 14 (Figures 2F,H). There was no significant difference between the cohorts with respect to relative expression of either gene within the middle ear fluids at any time point assessed. A similar reduction of gene expression was observed within the mucosal biomass from day 2 to day 5 after challenge. However, at both days 5 and 14 post-challenge, cells from the mucosa tended to express more MPO and ELANE when challenged with the *modA2* ON variant compared to the *modA2* OFF variant. This difference was significant for MPO at day 14 post-challenge (Figure 2G).

### Activation of Innate Immune Cell Populations

**Figure 3:**
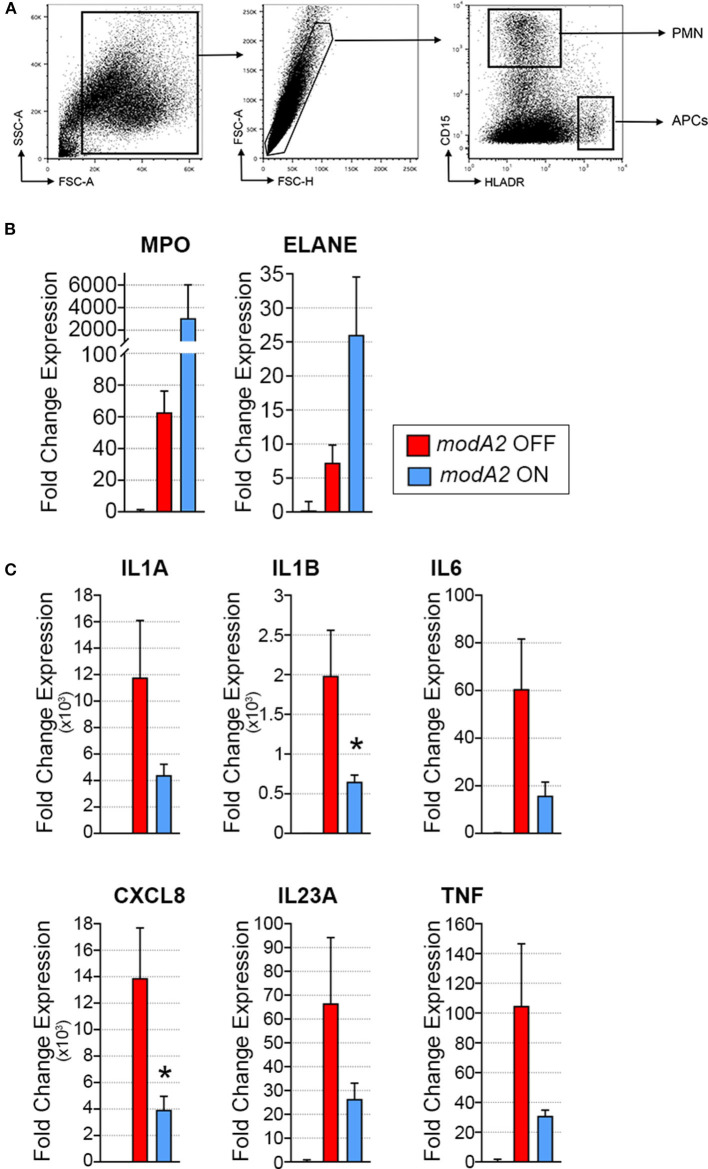
Gene expression of fluorescently sorted immune cells collected day 14 post-challenge. **(A)** APCs (CD15– HLADR+) and neutrophils (CD15+ HLADR–) were collected from the total middle ear (fluid + biomass) cell population. **(B)** Fold change gene expression of neutrophils, relative to naïve middle ear mucosa. Expression of myeloperoxidase, MPO, and neutrophil elastase, ELANE, was more induced by *modA2* ON compared to *modA2* OFF. **(C)** Fold change gene expression of APCs isolated from the middle ear, relative to naïve middle ear mucosa. Infection with *modA2* OFF induced greater expression of pro-inflammatory cytokines than infection with *modA2* ON. *n* = 3 ears. *p < 0.1, unpaired *t*-test.

Due to the heterogeneous nature of cell populations within the middle ear, we sought to assess gene expression of the individual APCs and neutrophils within the diseased ear. To this end, live cells collected from middle ears 14 days after challenge were sorted based on expression of cell surface markers, as indicated in Figure 3A. It is important to note that in contrast to previous experiments in which middle ear fluids and mucosal biomass were kept separate, subpopulation sorting was performed on total cells collected from the middle ear, which included cells from both the middle ear fluids and mucosal biomass. In agreement with the analysis of total mixed cell populations shown in Figure 2, CD15+ neutrophils from ears that contained the *modA2* ON variant expressed more relative MPO and ELANE that those from ears with the *modA2* OFF variant (Figure 3B). Interestingly, CD15- HLADR+ APCs from ears challenged with the *modA2* OFF population had significantly greater expression of the pro-inflammatory genes assessed compared to the those from ears challenged with *modA2* ON (*p* = 0.003, variation due to *modA2* status by 2-way ANOVA) (Figure 3C). These results suggested that the *modA2* OFF variant caused a greater APC mediated pro-inflammatory response compared to the *modA2* ON variant, and that the *modA2* ON variant induced a greater neutrophil response within the middle ear compared to the *modA2* OFF variant.

### Response of Chinchilla Neutrophils

**Figure 4:**
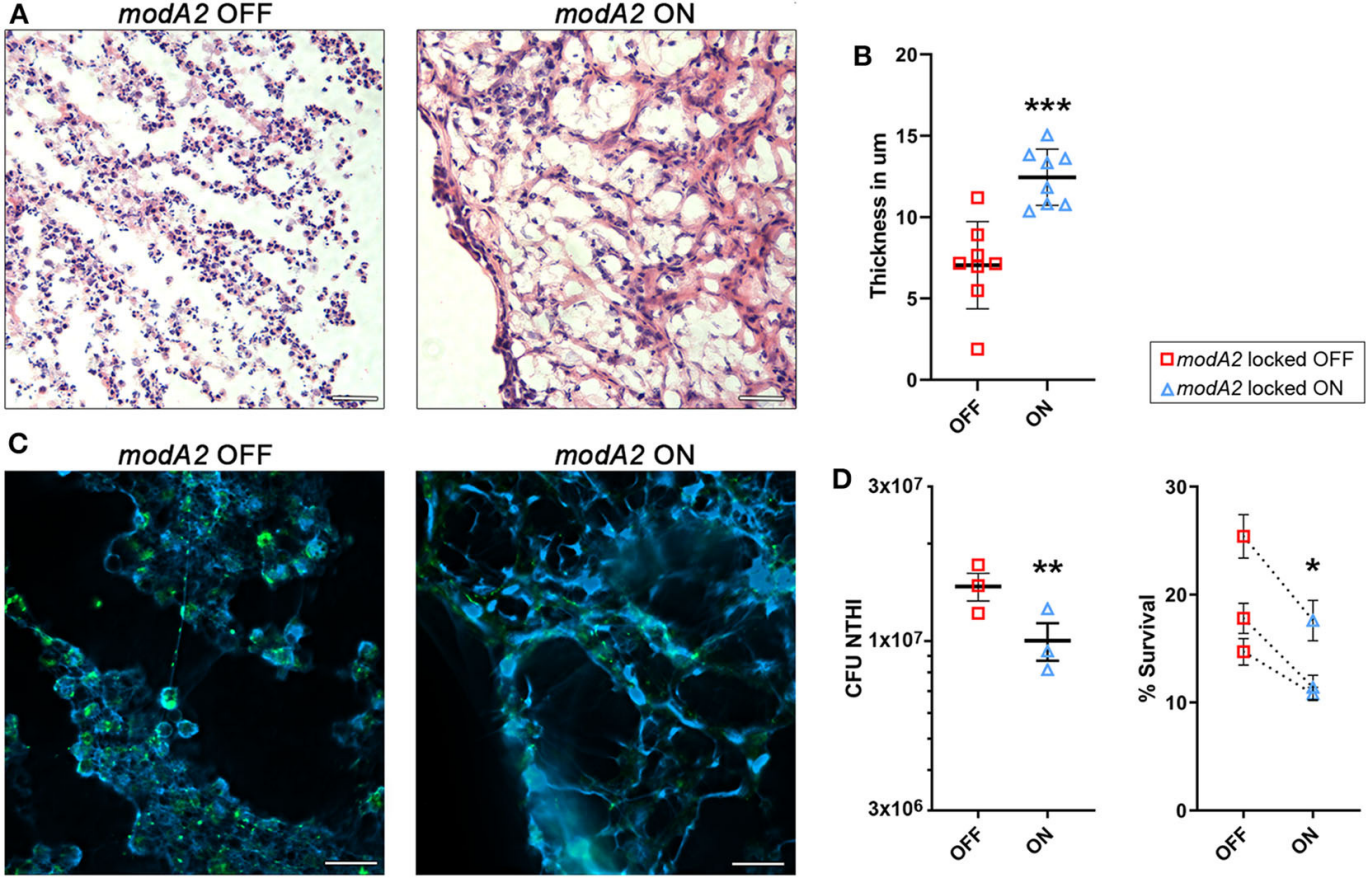
ModA2 phasevarion status affects neutrophil response and pathology within the chinchilla middle ear during disease. **(A–C)** Histological analysis of biomass collected from the chinchilla middle ear 14 days after challenge with *modA2* OFF or *modA2* ON. **(A)** Representative images of 5 μm sections stained with hematoxylin and eosin. Scale bar = 200 μm. **(B)** Average thickness of strand-like structures observed on the H&E stained sections. Symbols represent the average thickness measured across the field of view at an objective magnification of 63×. ***p < 0.001, unpaired *t*-test. **(C)** Representative images of 5 μm sections fluorescently labeled for DNA (blue) and bacteria (green). Scale bar = 20 μm. **(D)** Chinchilla neutrophils were infected with *modA2* OFF or *modA2* ON at a MOI of 10. After 45 min, extracellular bacteria were removed, and the cells were incubated for an additional 3 h to assess intracellular killing. Left panel, significantly more viable bacteria were collected from neutrophils infected with *modA2* OFF compared to those infected with *modA2* ON. Right panel, percent survival of NTHi after incubation with chinchilla neutrophils. Each point represents the average survival for each of three individual experiments with neutrophils isolated from different chinchillas, replicate values for each chinchilla are shown in Supplementary Figure 4. Dashed lines indicate paired experiments. *p < 0.05, **p < 0.01, paired-*t*-test.

Due to the fact that neutrophils are highly recruited to the middle ear during NTHi-induced infection in the chinchilla, we compared the histopathology of infection when chinchillas were challenged with the *modA2* OFF or *modA2* ON variants. Middle ear biomass from chinchillas infected with *modA2* OFF or *modA2* ON for 14 days were stained with hematoxylin and eosin (H&E) (Figure 4A). Abundant neutrophils were observed in both groups, forming large strand-like structures and clumps. Microscopic images were analyzed to assess the structure of the biomass collected from within the middle ear. Cell density and average thickness of the strand-like structures were blindly scored (scoring criteria are provided as Supplementary Table 1). No significant difference in host cell abundance and density was observed between cohorts, however ears challenged with *modA2* ON had significantly thicker strand-like structures than those challenged with *modA2* OFF (Supplementary Figure 3). To confirm these results, the average width of the strand-like structures was determined using Zeiss Zen 3.0 software. The strand-like structures formed within the ears challenged with *modA2* ON were significantly thicker than those challenged with *modA2* OFF (12.45 μm vs. 7.05 μm, *p* = 0.0003, unpaired *t*-test; Figure 4B).

As neutrophils can contribute to the establishment of biofilms by producing Neutrophil Extracellular Traps (NETs), we assessed this possibility by performing immunohistochemical analysis of unfixed tissues collected from the middle ear at day 14 post-infection. Interestingly, both strains of NTHi induced the formation of NET-like structures, but those structures were more abundant in the chinchillas challenged with *modA2* ON (Figure 4C). Animals infected with *modA2* OFF appeared to have more bacterial aggregates, visualized as larger areas of bacterial fluorescent labeling (Figure 4C, green), than those infected with *modA2* ON. Future work will be necessary to define the exact structure and composition of the bacterial aggregates observed.

To determine if the observed *in vivo* responses of innate immune cells to each variant were specific or a result of the larger inflammatory environment within the middle ear, we assessed neutrophil responses *in vitro*. Neutrophils were isolated from unchallenged, naïve chinchillas and co-cultured with NTHi *modA2* locked populations at a MOI of 10 NTHi per neutrophil. Neutrophils were allowed to phagocytose the NTHi for 45 min and then incubated for 3 h to assess bacterial survival within the neutrophils. Significantly more viable *modA2* OFF were recovered from within the chinchilla neutrophils compared to *modA2* ON recovered (p = 0.008, paired t-test) (Figure 4D, left). Percent survival was almost 50% greater with the *modA2* OFF compared to *modA2* ON population (19.6% vs 13.3%, *p* = 0.031, paired *t*-test; Figure 4D, right). Replicate values for each of the individual chinchilla are provided in Supplementary Figure 4.

### Response of Human Phagocytic Cells to NTHi *modA2* Variants

**Figure 5:**
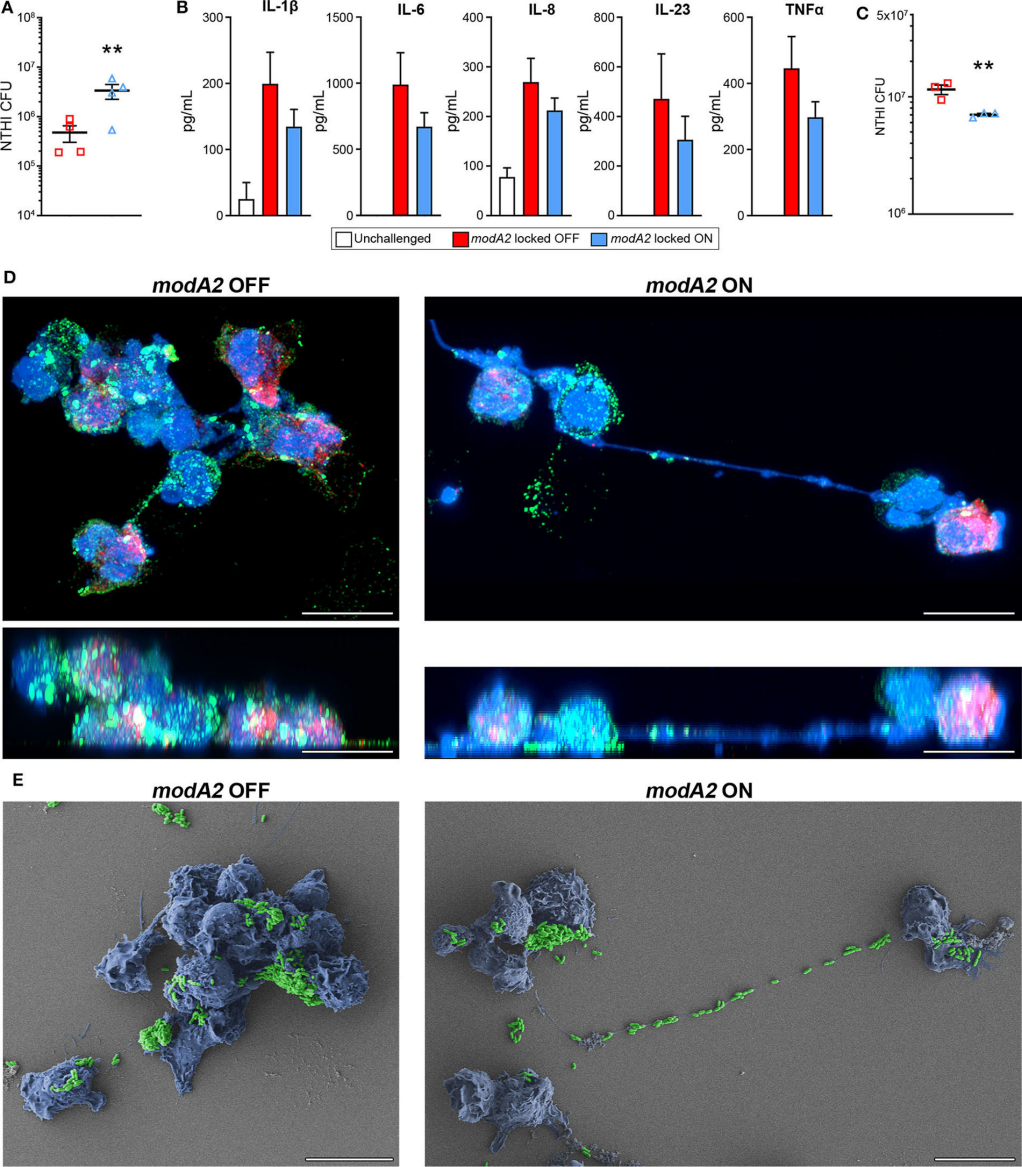
Response of human innate phagocytic cells to *modA2* OFF and *modA2* ON. **(A)** Human monocyte derived macrophages (hMDMs) were infected with *modA2* OFF or *modA2* ON at a MOI of 10. Viable NTHi, colony forming units (CFU), were enumerated 16 h post infection. Significantly more viable bacteria were recovered from the *modA2* ON population compared to the *modA2* OFF population. **(B)** Accumulation of soluble cytokines in the growth medium 16 h post infection was measured by cytokine bead array. Infection with *modA2* OFF induced greater expression of pro-inflammatory cytokines than infection with *modA2* ON *n* = 4. **(C)** Human peripheral neutrophils were infected with *modA2* OFF or *modA2* ON at a MOI of 10. After 45 min, extracellular bacteria were removed to assess intracellular killing. Significantly more *modA2* OFF were recovered compared to *modA2* ON. Replicate values for each donor shown in Supplementary Figure 4. **(D)** Representative renderings of neutrophils infected with *modA2* OFF or *modA2* ON for 3 h. Shown as top down (upper) and side (lower) views of 3D image stack. NTHi = green, DNA = blue, neutrophil elastase = red. Scale bar = 10 μm. Neutrophils infected with *modA2* OFF formed clumps of neutrophils filled with bacteria. Neutrophils infected with *modA2* ON released neutrophil extracellular traps (NETs) that captured the extracellular bacteria. **(E)** Scanning electron micrographs of neutrophils infected with *modA2* locked variants for 3 h. NTHi are pseudo colored green, neutrophils are pseudo colored blue. Micrographs of neutrophils from two additional donors are shown in Supplementary Figure 5. Scale bar = 10 μm. Images represent a minimum of triplicate studies. **p < 0.05, paired *t*-test.

In order to compare the innate immune response of the chinchilla phagocytic cells with that of humans, we next assessed the response of human monocyte-derived macrophages (MDMs) and human blood neutrophils to the *modA2* locked OFF and *modA2* locked ON variants. First, we assessed the ability of MDMs to recognize and kill each *modA2* variant. NTHi locked variants were added to MDMs at a MOI of 10 and incubated for 16 h. After 16 h, significantly more viable *modA2* ON remained compared to viable *modA2* OFF (Figure 5A). Soluble cytokine concentrations in the growth medium were measured and, as expected, cytokine production increased when MDMs were exposed to either variant compared to uninfected controls. MDMs exposed to the *modA2* OFF variant had greater concentrations of the pro-inflammatory cytokines, IL-1β, IL-6, IL-8, IL-23, and TNFα compared to those exposed to the *modA2* ON variant (Figure 5B). The increase observed was consistent across the cytokines tested but was not significant for any individual cytokine, which may have been due to the extended (16 h) incubation before measurement. In any case, the trend observed with the human MDMs paralleled the cytokine expression of APCs collected from the chinchilla middle ear (compare Figures 5B, 3B). While a combination of host cell and bacterial factors likely contribute to these observations, these data suggest that the *modA2* OFF variant may cause greater activation and increased expression of pro-inflammatory cytokines by macrophages, which in turn resulted in greater killing of this variant by macrophages.

We have shown previously that the *modA2* ON variant is more susceptible to killing by neutrophil-derived reactive oxygen species than the *modA2* OFF variant (Brockman et al., 2017). Here we assessed the ability of human neutrophils to recognize and kill each individual variant. To assess internalization and killing, NTHi were added to unstimulated neutrophils for 45 min and then the extracellular bacteria were removed. After an additional 3 h of incubation, significantly less viable bacteria remained from the *modA2* ON variant compared to the *modA2* OFF variant (Figure 5C). Replicate values for each donor are provided in Supplementary Figure 4. Differences in recognition and uptake of the variants by neutrophils and relative survival of the phagocytized bacteria likely contribute to the fate of each of these populations. To identify potential differences in neutrophil response to each population, neutrophils were exposed to each *modA2* variant for 3 h, then fixed and imaged by confocal microscopy and scanning electron microscopy. Substantial differences in neutrophil morphology were apparent between those exposed to the *modA2* OFF variant and those exposed to the *modA2* ON variant. Neutrophils exposed to *modA2* OFF exhibited considerable clumping (DNA shown in blue) and had aggregates of viable bacterial (shown in green) within and on the surface of the neutrophils (Figure 5D, left panels). Large bacterial aggregates (or biofilms) (green) were also observed on the surface of neutrophil clumps (blue) when visualized by electron microscopy (Figure 5E, left panel). In contrast, neutrophils exposed to the *modA2* ON variant displayed long extracellular DNA strands (shown in blue) indicative of NETosis (Figure 5D, right panel). Viable *modA2* ON bacteria (shown in green) were localized to the surface of some neutrophils (Figure 5D, right panel). Some aggregates of *modA2* ON were also observed on the surface of neutrophils by electron microscopy (Figure 5E, right panel). Of particular note, many bacteria appeared to be caught within the extracellular DNA that was released from the neutrophils, yet very few viable bacteria, based on GFP signal, were found associated with the extracellular DNA when observed by confocal microscopy (compare Figures 5D,E, right panels). Representative micrographs from two additional donors are shown in Supplementary Figure 5. These data suggest that the *modA2* ON variant induces the production of NETs that are able to efficiently capture and kill the bacterial cells.

## Discussion

Phase variable regulons, or phasevarions, have been identified in multiple bacteria associated with the human respiratory and gastrointestinal tracts (Srikhanta et al., 2005, 2009, 2011, 2017a; Blakeway et al., 2014). Many of these are pathobionts that asymptomatically colonize portions of the human respiratory tract, such as the nasopharynx, but are also capable of causing disease throughout the airways. Previous work has shown that the ModA2 phasevarion of non-typeable *Haemophilus influenzae* (NTHi) affects the progression and severity of experimental otitis media, and that a shift in phasevarion status within the middle ear results in significantly greater disease severity that when a shift does not occur (Brockman et al., 2016). In this study, we used variants of NTHi strain 723 in which *modA2* has been genetically locked and thus is unable to phase vary or shift status within the middle ear. The use of these non-switching variants allowed us to first determine the unique host response specific to each of the individual subpopulations, *modA2* OFF or *modA2* ON. NTHi strain 723 was selected because it is a pediatric otitis media isolate that represents the predominate *modA* allele (*modA2*) across multiple clinical NTHi collections and is also the most studied NTHi phasevarion isolate to date (Atack et al., 2015a, 2019b). The findings presented here using variants in which *modA2* is unable to phase vary will serve as the foundation for studies to investigate host response when *modA* is free to phase vary, as well as with other important phase variable *modA* alleles.

Several experimental models of otitis media have been developed over the past few decades and each present a unique set of benefits and limitations. The most commonly used animal models are the chinchilla, rat and mouse, with transbullar delivery, directly into the middle ear space, the most used route of pathogen administration (Davidoss et al., 2018). Chinchillas have the advantage of large bullae that are anatomically and histologically similar to the human middle ear. They also rarely develop spontaneous OM but are susceptible to human otopathogens. However, relatively few chinchilla-specific tools are commercially available, which has limited in-depth immunological studies to this point. It is important to note that chinchillas used for research are outbred and thus better represent the genetic diversity found within the human population, and as such have been the gold standard for pre-clinical studies of treatment and vaccine strategies. The middle ear anatomy and histology of rats is also comparable with that of humans and the rat middle ear is relatively large. More reagents are commercially available for rats, but the inbred nature of research strains limits some of the translational power of this model for pre-clinical studies. Mice are the most commonly used model to study immune response during OM due to the relatively low cost and wide range of commercially available reagents and knock-out strains. In addition, a Junbo mutant mouse that develops spontaneous otitis media has provided further insight into immune responses during otitis media (Hood et al., 2016; Vikhe et al., 2019a,b). Herein, we developed new chinchilla-specific reagents and utilized innovative techniques to begin to assess host immune responses in the chinchilla model of NTHi-induced otitis media. We used a variety of fluorescent flow cytometry and gene transcription assays to show that regulation by the ModA2 phasevarion of NTHi dictates differential immune responses to infection within the middle ear.

Disease progression following challenge with either of the NTHi strain 723 *modA2* locked variants, used in this study, was similar to our previously published work with phase variable, non-locked, strains that did not shift status (Brockman et al., 2016). In all studies, outcomes of experimental diseases were significantly more severe when the population shifted from *modA2* OFF to *modA2* ON, compared to strains that either did not shift or locked strains that were unable to shift (Brockman et al., 2016). There were no significant differences in the amount of middle ear biomass or fluids present 2 or 5 days after challenge with *modA2* locked OFF or *modA2* locked ON. Although, the bacterial burden recovered at day 14 after challenge in middle ears was similar in chinchillas infected with *modA2* ON or *modA2* OFF, animals challenged with *modA2* ON had significantly more mucosal biomass and fluid within the middle ear compared to those challenged with *modA2* OFF. Middle ears challenged with *modA2* ON also had significantly more antigen presenting cells (APCs) and neutrophils at day 14 post infection, compared to those challenged with *modA2* OFF. These differences may be due to subtle and yet-unidentified differences in the early stages of infection. It is important to note that only viable cells were included in the enumeration of immune cell populations by flow cytometry and that these numbers likely underrepresent the total immune cell infiltration, due to cell death pathways (i.e., NETosis or necrosis), and this may explain discrepancies between scarce counts of early recruiting neutrophils, which did not correlate to increased expression of MPO or ELANE in the middle ear of infected animals at 2 day post infection. Neutrophil extracellular traps (NETs) are an important part of the immune response to bacterial infection and are present within the infected middle ears of chinchillas and humans (Schachern et al., 2017). Multiple stains of NTHi have been shown to activate neutrophil response and induce the release of neutrophil extracellular traps (NETs). Lipooligosaccharides (LOS) and to lesser degree, bacterial outer membrane proteins (OMPs) and DNA, act as signals to induce the production of NETs (Juneau et al., 2011). Furthermore, NETs contribute substantially to the overall biofilm biomass within the diseased middle ear, which in turn contributes to increased bacterial survival and persistence (Hong et al., 2009; Juneau et al., 2015). Neutrophil activation and response is also important in the pathogenesis of NTHi-induced infections throughout other regions of the human respiratory tract, including the lungs (Naylor et al., 2007; Storisteanu et al., 2017). The overall differences observed between challenge cohorts at day 14 may reflect a change in the initial innate immune response to infection or alteration in the early adaptive immune response, which remains to be tested. It is known that a shift in *modA2* status within the chinchilla middle ear results in significantly greater experimental disease severity (Brockman et al., 2016). While not directly tested in the studies herein, we hypothesize that the differences in response toward each individual variant (*modA2* ON or OFF) contribute to the increased disease severity observed when *modA2* status shifts and the host is thus exposed to both unique variants.

It is well-established that regulation by the ModA2 phasevarion alters the expression of numerous bacterial genes and, in turn, encoded virulence products. Due to the epigenetic nature of this regulation, it is difficult to determine the exact genes regulated by the phasevarion and the specific conditions under which they are regulated. However, as the *modA2* OFF and *modA2* ON variants each express a unique collection of virulence factors, we anticipated each may drive a unique immune response. To evaluate immune response and activation within the middle ear we first performed targeted studies on samples collected day 14 after challenge to determine gene expression of pro-inflammatory cytokines and neutrophil activation markers. No significant differences in expression were observed when we analyzed total RNA collected from within the middle ear, likely due to the tremendously heterogeneous nature of these samples (i.e., large number of different immune and epithelial cell types). However, substantial differences in gene expression were observed when distinct viable immune cell populations were assessed, following fluorescently-activated-cell-sorting. APCs, which likely include mononuclear phagocytic cells, isolated from the middle ears of chinchillas challenged with *modA2* OFF expressed significantly more pro-inflammatory cytokines than those that received *modA2* ON. These results indicated that *modA2* OFF, or a virulence factor expressed by this variant, induced greater activation or recognition by these phagocytic cells than did *modA2* ON. We hypothesized that most of these APCs were macrophages, as neutrophils and macrophages constitute the majority of immune cells that infiltrate the middle ear early during otitis media (Hernandez et al., 2015; Kurabi et al., 2015). Furthermore, *modA2* OFF also lead to a greater overall level of pro-inflammatory cytokine production by human monocyte-derived macrophages (hMDMs) during a 16-h *in vitro* assay. Interestingly, the *modA2* OFF variant was significantly more sensitive to killing by hMDMs than the *modA2* ON variant, which may be due to greater activation of the macrophages in the presence of *modA2* OFF compared to *modA2* ON. Future work is necessary to fully investigate the mechanisms by which macrophages differentially recognized and kill each of these populations. Many proteins and saccharides on the outer surface of NTHi are known to affect opsonization and host immune response and may be regulated by ModA2 during disease (Duell et al., 2016; Winter and Barenkamp, 2017; Li et al., 2018). The potential ability of the *modA2* ON subpopulation to avoid recognition and/or suppress activation by macrophages could result in increased survival and a subsequent shift toward a predominantly *modA2* ON population, such as the one observed within middle ear fluids during experimental disease (Atack et al., 2015a).

Neutrophils collected from the middle ears of chinchillas challenged with *modA2* ON expressed more myeloperoxidase (MPO) and neutrophil elastase (ELANE) compared to those challenged with *modA2* OFF, suggesting that *modA2* ON recruited more neutrophils to the middle ear. Catalase and peroxiredoxin-glutaredoxin enzymes produced by NTHi are protective against oxidative stressors and can promote bacterial persistence within NETs (Juneau et al., 2015). In combination with biofilm formation, increased oxidative stress resistance and greater survival within NETs have been shown to contribute to NTHi persistence within the chinchilla middle ear (Hong et al., 2009). Intriguingly, the *modA2* OFF variant is more resistant to neutrophil-derived reactive oxygen species (ROS) than the *modA2* ON variant, due to increased resistance to the antimicrobial effects of hydrogen peroxide (Brockman et al., 2017). However, previous work only investigated the effect of the ModA phasevarion on NTHi resistance to neutrophil-derived ROS, but did not directly investigate the interaction of the bacteria with unstimulated neutrophils (Brockman et al., 2017). In the current study, we began to investigate potential differences in the recognition and response of neutrophils to the *modA2* locked OFF or *modA2* locked ON variants. Expression data from neutrophils within the middle ear suggested that *modA2* ON induce greater activation, which could result in increased production of ROS and NETs, and ultimately greater bacterial killing. Indeed, using *in vitro* assays with unstimulated chinchilla and human neutrophils we found that significantly less viable bacteria were recovered when neutrophils were challenged with *modA2* ON compared to *modA2* OFF. It is likely that *modA2* ON expresses different, yet to be identified, Pathogen-Associated Molecular Pattern (PAMPs), such as LOS or OMPs, than *modA2* OFF, which could induce a differential activation of neutrophils. Several NTHi virulence factors can exhibit phase variable expression independent of the ModA2 regulon (Poole et al., 2013; Atack et al., 2015b; Phillips et al., 2019). While both variants were identical at time of inoculation, with the exception of the *modA2* repeat region, it is possible that ModA2-independent phase variation occurred and may contribute to some of the heterogeneity in disease. Several genes in the LOS biosynthesis pathways are known to undergo phase variation, independent of *modA* (Phillips et al., 2019). Future work to assess the role of specific virulence factors in immune response must consider both ModA-dependent and -independent phase variation. Given the fact that NTHi forms biofilms within the middle ear, future work should also address the impact of the ModA phasevarion on the ability of neutrophils and NET formation to prevent or enhance the establishment of bacterial persistence that results in a chronic inflammatory process. Both, chinchilla and human neutrophils showed similar results in the antimicrobial mechanisms against NTHi variants, being more efficient at killing *modA2* ON compared to *modA2* OFF. Because the study of cellular and molecular mechanisms of cells-derived from chinchilla present additional technical challenges, here we also show that human and chinchilla phagocytes can be used as a comparative model to study the interaction between the immune response and NTHi.

Taken together, the results of the *in vitro* experiments suggest that the phasevarion can regulate the response of innate immune cells to bacterial infection. It is interesting how epigenetic regulation of bacterial gene expression, due a single phase variation event, may lead to such a stark difference in the response of these two innate immune cell populations, neutrophils and macrophages. As the immune response evolves over the course of disease, the ability to shift between these two different variant phenotypes likely provides an advantage for the survival and persistence of the total NTHi population. Work to better understand the specific intricacies of these interactions is needed; however, it is likely that the ModA2 phasevarion alters the expression of bacterial virulence factors, which in turn changes the way immune cells recognize and respond to infection. It is also possible that regulation by the phasevarion affects the way NTHi sense and respond to their host environment, which would correspondingly change bacterial response and expression of genes not directly regulated by ModA2 itself. Irrespective of the mechanisms involved, we have shown that regulation by the phasevarion alters immune responses to this bacterial pathogen.

This work provides a basis to better understand the complex interactions of the NTHi phasevarion and phagocytes within the middle ear during disease. We also reveal for the first time the importance of the ModA2 phasevarion in the alteration of innate immune responses of the host and that *modA2* status significantly impacts the nature and effectiveness of innate immune responses. While *modA2* represents the predominant *modA* allele identified to date, future work is necessary to fully understand the role of other phase variable *modA* alleles in host immune response and disease. The work presented here provides a basis for studies to investigate the role of other ModA phasevarions in the regulation of innate immune responses and disease severity. Furthermore, this unique bacterial regulation of host immune response due to the phasevarion may have implication in the treatment and prevention of not only NTHi-induced otitis media, but infections caused by other mucosal pathogens that employ a phase variable regulon.

## Methods

### Bacterial Strains and Culture Medium

NTHi strain 723 was received from the Finnish Otitis Media study group (Meats et al., 2003). NTHi strain 723 variants in which *modA2* phase variation is locked, and bacteria are unable to switch status, were generated previously (Brockman et al., 2017). Generation of strain 723 *modA2* locked variants that constitutively express GFP was described previously (Brockman et al., 2017). NTHi were routinely cultured in brain heart infusion broth supplemented with hemin (2 μg/mL) and NAD (2 μg/mL) or on chocolate agar and grown at 37°C with 5% CO_2_.

### Chinchilla Model of NTHi-Induced Otitis Media

Adult chinchillas (*Chinchilla lanigera*) weighing 500–800 g were obtained from Rauscher's Chinchilla Ranch (LaRue, OH) and acclimated in the vivarium for 7 days. Serum was collected from chinchillas prior to bacterial challenge to obtain baseline pre-immune serum. Chinchillas were cohorted based on sex and weight, and then challenged by transbullar inoculation with ~750 CFU of NTHi in 300 μL of saline/bulla to induce experimental otitis media. At the time of challenge, the number of NTHi CFU per milliliter was confirmed by serial dilution and plating of inocula. At days 2, 5, and 14 after bacterial challenge, a pre-determined group of anesthetized chinchillas were euthanized, and the bullae were dissected from the skull. Left and right bullae were aseptically opened and imaged. Middle ear fluid and middle ear mucosa plus any adherent biomass were collected from the left ears for subsequent immunologic analyses. Fluids and mucosal samples from the right ears were collected and used for subsequent RNA isolation. The volume and weight of the respective samples were recorded at time of collection and before any processing.

All animal experiments were performed under protocol AR17-00073 approved by the Institutional Animal Care and Use Committee at the Research Institute at Nationwide Children's Hospital or protocol AUA00006766 approved by the Institutional Animal Care and Use Committee at the Medical College of Wisconsin, and in accordance with the National Institutes of Health Guide for the Care and Use of Laboratory Animals.

### Gross Mucosal Biofilm Scoring

To assess biofilm formation within the middle ear, images of each middle ear were scrambled and then blindly scored by a minimum of 6 experienced reviewers on a 0–4 scale, wherein 0 indicated no biofilm, 1 indicated that biofilms filled <25% of the middle ear, 2 indicated that biofilm filled 25–50% of the middle ear, 3 indicated that biofilm filled 50–75% of the middle ear, and 4 indicated that biofilm filled >75% of the middle ear (Goodman et al., 2011). Data represent the average of all reviewers for each image.

### Microscopic Analysis of Middle Ear Biomass

To assess the structure and composition of the middle ear biomass chinchillas were challenged as described above using NTHi strain 723 *modA2* locked variants that constitutively expressed GFP. After 14 days a small amount of biomass was excised and snap frozen in Tissue-Plus O.C.T. compound (Fisher Scientific). Frozen sections (5 μm thick) were either processed for standard hematoxylin and eosin (H&E) staining or fixed and used for fluorescent immunohistochemistry (IHC). For IHC, the sections were probed with mouse anti-dsDNA antibodies (Abcam) and DyLight680-conjugated anti-GFP antibodies (ThermoFisher), the slides were washed twice and the anti-dsDNA antibodies were fluorescently labeled with AlexaFluor 488-conjugated goat anti-mouse IgG (H+L) secondary antibodies (ThermoFisher). Images were captured on a Zeiss Axio Observer inverted fluorescent microscope and processed with Zeiss Zen 2.6. For H&E stained section, images of replicate fields of view of multiple different sections were imaged on a Zeiss AxioScope 5 microscope with a 63x oil objective and captured on Zen 3.0 (Zeiss). Scrambled images were blindly scored by at least 3 independent reviewers to assess immune cell density and thickness of the strand-like structures using the rubric provided in Supplementary Table 1. Following scoring, the thickness of all strands visible within each field of view were measured on Zeiss Zen 3.0 and the average of all measurements per field of view was recorded.

### Analysis of Immune Cell Composition Within the Middle Ear

Samples collected from the left ears were gently centrifuged and cell-free supernatants were snap frozen and stored. Cells were then resuspended in sterile saline and treated with DNAse and collagenase for 1 h and subsequently filtered with a 70 μM mesh, enumerated and divided for cell specific analysis. Washed cells were stained with the antibodies Alexa Fluor 700 anti-mouse/human CD11b clone M1/70 (Biolegend), biotin anti-human CD15 clone HI98 (Biolegend), BV 421 anti-human HLADR clone G46-6 (BD Horizon), FITC anti-mouse/human CD45R/B220 clone RA3-6B2 (Biolegend), APC-streptavidin (Biolegend), and incubated in ice-cold FACS buffer for 15 min, and analyzed on an LSR II cytofluorometer (Becton Dickinson, BD). Cells were gated according to size (SSC-A) and forward scatter (FSC-A) to obtain cell singlets and blue-fluorescent reactive dye L23105 (Life Technologies) was used to discard dead cells. Absolute cell numbers were calculated with the total cell count multiplied successively by the percentages for the appropriate gates obtained through flow cytometry.

### Fluorescence-Activated Cell Sorting of Specific Cell Populations

Following homogenization and enumeration, a fraction of cells collected on Day 14 were stained with the monoclonal antibodies anti-human BV421 HLADR, biotin anti-human CD15 and APC-streptavidin, then the cells were sorted on an Influx FACS (BD Biosciences, San Jose, CA) based on fluorescence profiles, as indicated. Due to the number of samples collected and sorter limits, cells from the fluid and mucosal samples of each ear were pooled prior to sorting at a ratio that reconstituted that present at time of collection. The resultant cell populations were homogenized in TRIzol reagent (ThermoFisher Scientific) and stored at −80°C for subsequent analysis.

### Quantitative Realtime PCR

Quantitative real-time reverse transcriptase PCR was performed to determine the relative expression of select chinchilla genes. Custom TaqMan Gene Expression Assays were designed and validated for chinchilla genes of interest. DNA-free RNA was isolated and purified using TRIzol reagent according to manufacturer instructions. cDNA libraries were subsequently generated with the High-Capacity RAN-to-cDNA Kit (Applied Biosystems). Quantitative PCR was carried out with TaqMan Advanced 2x Master Mix on an ABI 7500 real-time thermocycler (Applied Biosystems). Relative expression (transcript) was calculated by the delta-delta-Ct method (Livak and Schmittgen, 2001). Samples were normalized to 18s rRNA transcript and relative fold change in expression was calculated relative to middle ear mucosa from naïve unchallenged chinchillas. Relative levels of GAPDH and 18s rRNA transcript were assessed to verify RNA/cDNA integrity and similar results were obtained when normalized to 18s or GAPDH.

### Chinchilla Neutrophil Response

Chinchilla neutrophils were isolated from either peripheral blood or bone marrow collected from the femur and tibia of healthy, unchallenged animals. Cells from each animal were maintained distinctly and there was no pooling of samples between individual animals. Neutrophils were isolated by sequential rounds of density gradient centrifugation and visually confirmed following Kwik-Diff stain. Fresh neutrophils were incubated with NTHi at a MOI of 10 NTHi per neutrophil for 45 min at 37°C to allow phagocytosis and internalization. Non-internalized bacteria were then removed and 5 × 10^5^ neutrophils in 500 uL medium were seeded into the wells of 24-well plates. The infected neutrophils were incubated for 3 h at 37°C to allow bacterial killing. The Neutrophils were treated with 0.1% Triton-X100 to permeabilize the cells and NTHi were enumerated by dilution and plating onto chocolate agar. Neutrophils were collected from 3 independent adult chinchillas, isolated and analyzed separately, and all assays were performed in 4 or 5 replicates for each donor. Data represent the mean for each donor. Percent survival was calculated as CFU NTHi survival/CFU NTHi at challenge. Replicate values by donor are shown in Supplementary Figure 4.

### Response of Human Innate Immune Cells

Human peripheral blood neutrophils and monocytes were purified by EasySep negative selection (STEMCELL Technologies) from newly drawn blood according to the manufacturer directions. Fresh neutrophils were incubated with NTHi at a MOI of 10 NTHi per neutrophil for 45 min at 37°C to allow phagocytosis and internalization. Non-internalized bacteria were then removed and 5 × 10^5^ neutrophils in 500 uL medium were seeded into the wells of 24-well plates. The infected neutrophils were incubated for 3 h at 37°C to allow bacterial killing. The neutrophils were treated with 0.1% Triton-X100 to permeabilize the cells and NTHi were enumerated by dilution and plating onto chocolate agar. Neutrophils were collected from 3 independent adult donors, isolated and analyzed separately, and all assays were performed in triplicate for each donor. Data represent the mean for each donor. Replicate values by donor are shown in Supplementary Figure 4.

Monocytes were differentiated to Monocyte-derived Macrophages (MDMs) by culturing them with RPMI media plus 10% FBS for 5 days, then MDMs were detached and seeded in 24 well plates at a density of 5 × 10^5^ cells per well a day prior to infection. The next day, MDMs were infected with NTHi at MOI of 10 for 45 min at 37°C. Extracellular bacteria were removed by washing the wells 3 times with PBS, and then cells were incubated at 37°C overnight. 0.1% Triton-X100 was added to release the intracellular bacteria and NTHi CFU were enumerated by performing serial dilutions on chocolate agar. Before addition of Triton-X100, a small aliquote of the cell culture medium was collected and total cytokines levels were measured with a Custom Cytometric Bead Array (BD Biosciences) analyzed on an Accuri C6 (BD Biosciences).

### Confocal Microscopy

A total of 10^5^ human neutrophils were seeded onto chambered coverglass for 30 min at 37°C, and then NTHi that constitutively express GFP (green fluorescent protein) were added at an MOI of 10 and incubated for an additional 3 h at 37°C. Cells were fixed for 30 min in 10% neutral buffered formalin, stained with fluorescent monoclonal antibodies against neutrophil elastase, triple rinsed with PBS and the DNA was stained with DAPI. No additional NTHi-specific staining was used as GFP fluorescence is maintained within intact NTHi cells following fixation. Decreased membrane integrity resulted in loss of GFP signal, which correlated with cell death assessed by bacterial viability staining. The samples were imaged on a LSM 800 laser scanning confocal microscopy with Airyscan detector and images were rendered with Zeiss Zen v2.5.

### Scanning Electron Microscopy

A total of 10^5^ human neutrophils were seeded onto coverslips for 30 min at 37°C, and then NTHi were added at a MOI of 10 and incubated for an additional 3 h at 37°C. The cells were then fixed with 2.5% glutaraldehyde in PBS buffer for 30 min, followed by a second 30 min fixation with 1% osmium tetroxide in PBS. The fixed samples were then triple washed in deionized water and dehydrated with graded ethanol (50, 70, 80, 90, 95, and 100%). The ethanol was then replaced with hexamethyldisilazane (HMDS) and the samples were allowed to air dry. The coverslips were coated with 2.5 nM gold/palladium in an Emitech K550X sputter coater and examined on a Hitachi S-4800 field-emission scanning electron microscope. Images were captured at an accelerating voltage of 3–5 kV using a secondary electron detector and psuedocolored with Adobe Photoshop CC 2019. Human neutrophils were collected from 3 independent donors. Figure 5 shows representative images of neutrophils from donor 51, raw micrographs of neutrophils from donors 7 and 53 are presented in Supplementary Figure 5.

### Statistical Analysis

All statistics were performed with GraphPad Prism v 8.0, as indicated.

## Data Availability Statement

The datasets generated for this study are available on request to the corresponding author.

## Ethics Statement

The animal study was reviewed and approved by Institutional Animal Care and Use Committee at the Research Institute at Nationwide Children's Hospital and the Medical College of Wisconsin.

## Author Contributions

KB: conceptualization. FR-A, JR-R, and KB: methodology. FR-A, JR-R, and KB: investigation. KB and FR-A: writing—original draft. JR-R and SP-S: writing—review & editing. KB: funding acquisition. SP-S and KB: supervision. All authors contributed to the article and approved the submitted version.

## Conflict of Interest

The authors declare that the research was conducted in the absence of any commercial or financial relationships that could be construed as a potential conflict of interest. The reviewer DH declared a past co-authorship with one of the authors KB to the handling editor.
