# Supplementary material for: A Bacterial Epigenetic Switch in Non-typeable Haemophilus influenzae Modifies Host Immune Response During Otitis Media
Source: Front Cell Infect Microbiol. 2020 Oct 23;10:512743. doi: 10.3389/fcimb.2020.512743 (PMC7644868; doi:10.3389/fcimb.2020.512743)
Supplement: Supplementary file 1 [file Data_Sheet_1.pdf]

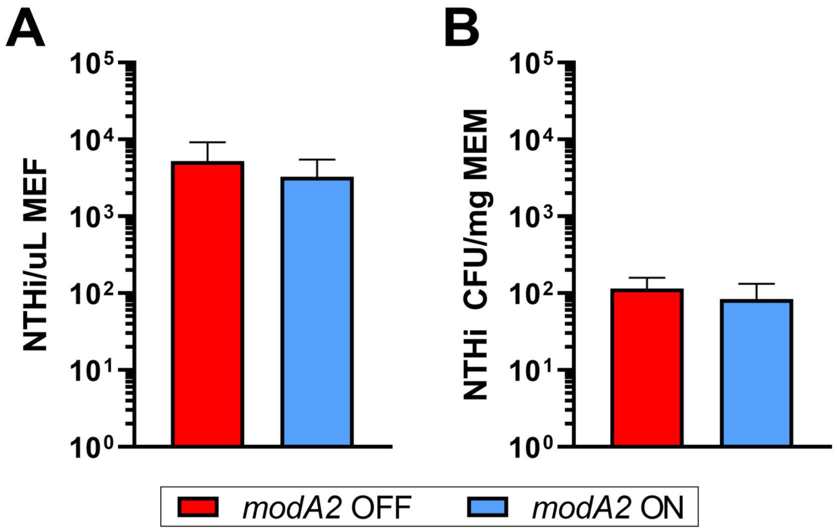

Supplementary Figure 1. Bacterial burden in the chinchilla middle ear 14 days after challenge. Viable NTHi in the middle ear fluids (A) and middle ear mucosa + adherent biomass (B) were enumerated. At 14 days post challenge there was no significant difference in the concentration of *modA2* OFF or *modA2* ON within the ear. Unpaired t-test, n = 6.

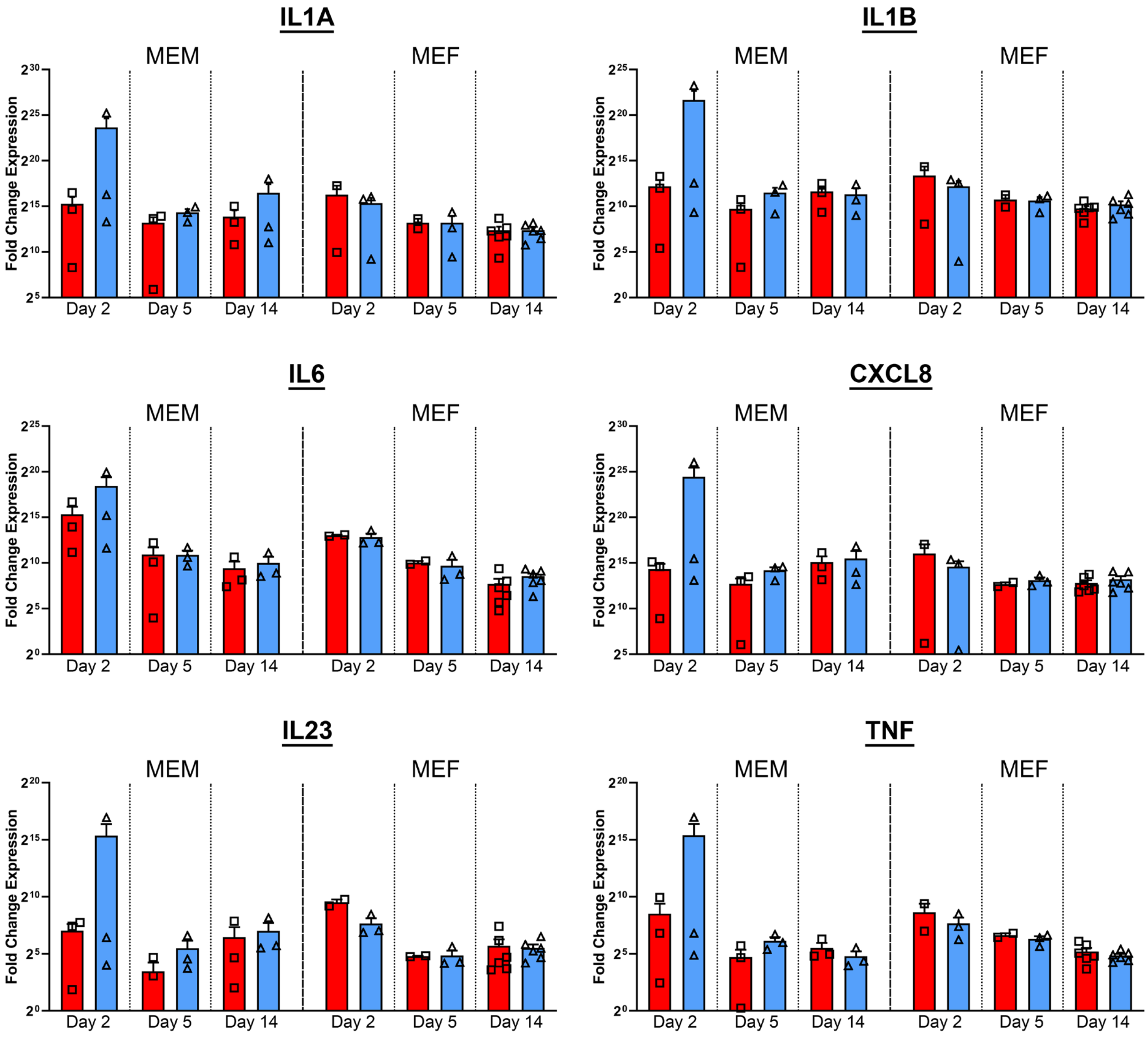

Supplementary Figure 2. Gene expression of immune cells collected from the middle ear during experimental disease.
Relative expression of select pro-inflammatory cytokine genes relative to uninfected chinchilla middle ear mucosa was determined by quantitative real-time RT-PCR. RNA was collected from the entire middle ear mucosa + biomass (MEM) or middle ear fluids (MEF). The expression of all gene targets was significantly greater in the infected compared to the non-infected ears. There were no significant differences between animals challenged with the *modA2* locked OFF (red bars) or the *modA2* locked ON (blue bars) variants.

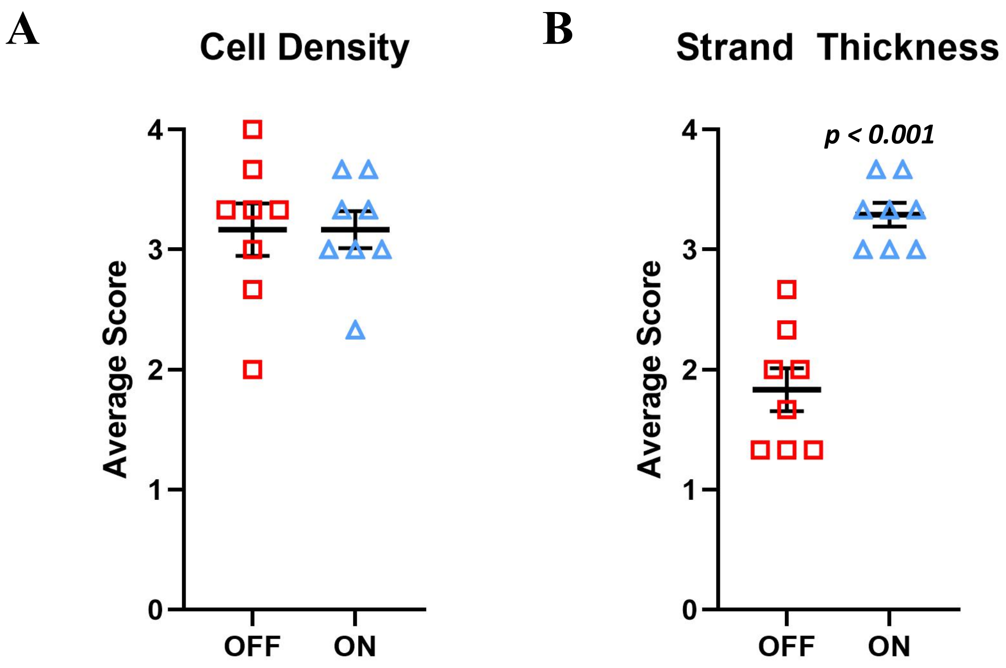

Supplementary Figure 3. Histological analysis of chinchilla middle ear biofilm biomass. Images of hematoxylin and eosin (H&E) stained sections of biofilm biomass collected from chinchilla middle ears 14 days after challenge were scored using the rubric in Supplementary Table 1. Individual fields of view were blindly reviewed by 3 individual reviewers and the average score for each sample is shown. (A) There was no significant difference in immune cell density between ears challenged with *modA2* OFF compared to those challenged with *modA2* ON. (B) Biomass within ears challenged with *modA2* ON had strand-like structures that were significantly thicker than those present in ear challenged with *modA2* OFF. Unpaired Mann-Whitney test.

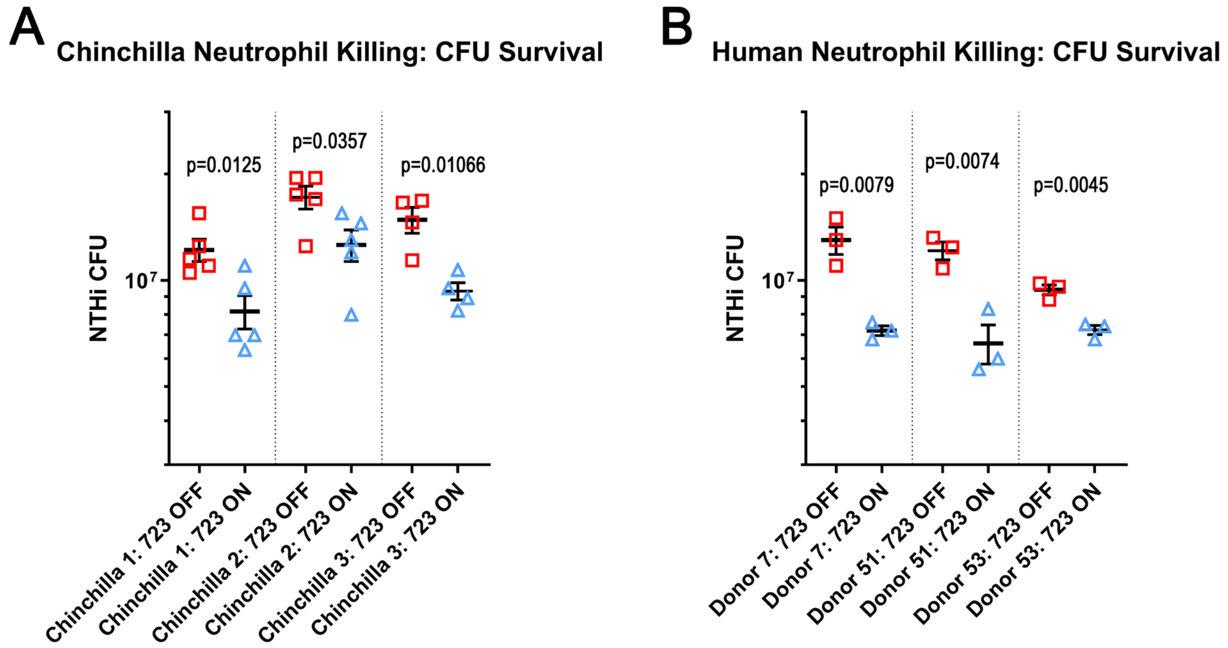

Supplementary Figure 4. Neutrophil killing by donor.
(A) Chinchilla neutrophils from three different naïve animals were infected with *modA2* OFF or *modA2* ON at a MOI of 10. Four or five independent replicates were performed for each donor. After 45 minutes, extracellular bacteria were removed to assess intracellular killing. For all donors, significantly more *modA2* OFF were recovered compared to *modA2* ON. Unpaired t-test.
(B) Human peripheral neutrophils from 3 different donors were infected with *modA2* OFF or *modA2* ON at a MOI of 10. Three independent replicates were performed for each donor and are indicated by each symbol. For all donors, significantly more *modA2* OFF were recovered compared to *modA2* ON. Unpaired t-test.

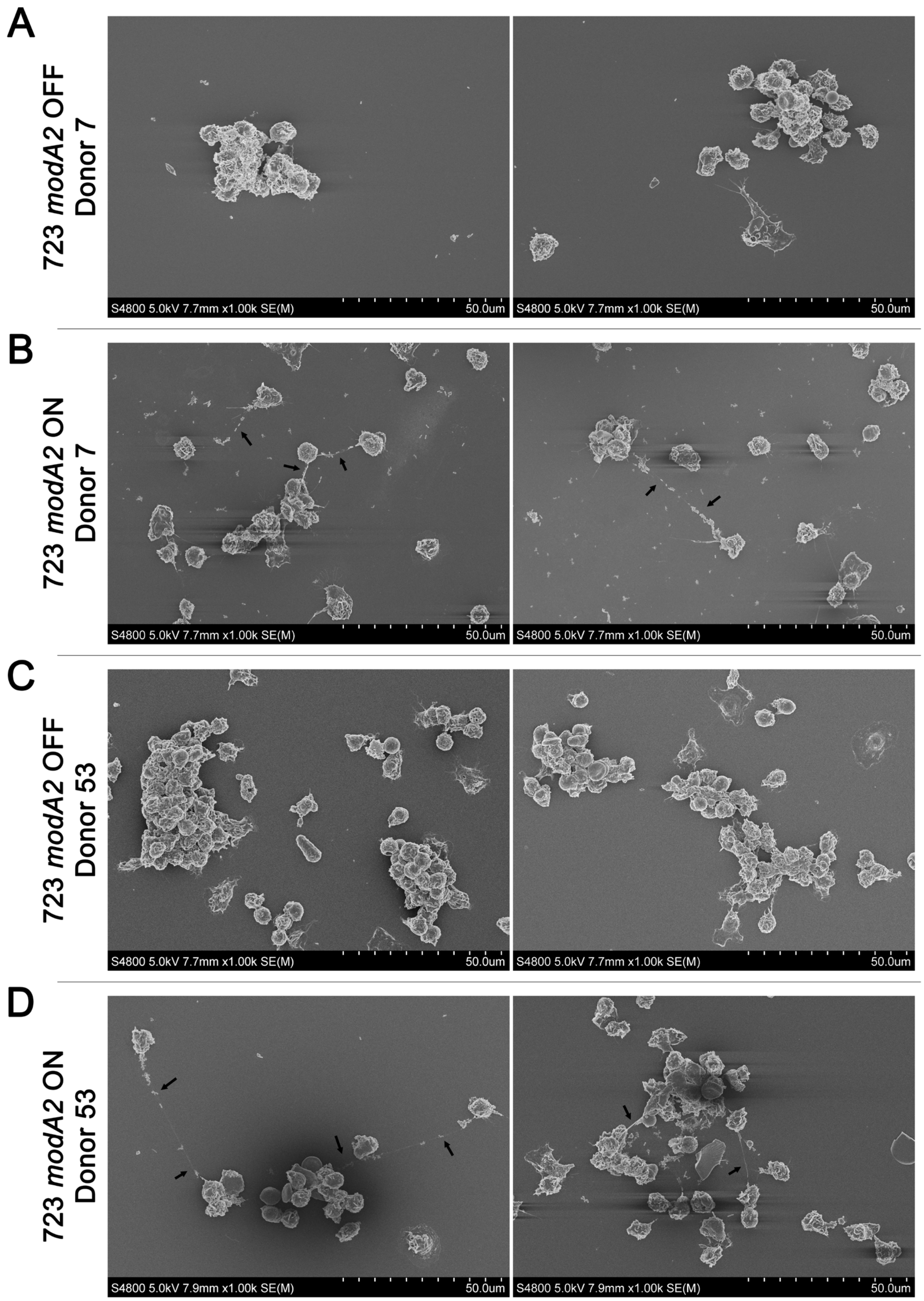

Supplementary Figure 5. Representative electron micrographs of NTHi challenged neutrophils. Scanning electron micrographs of human neutrophils infected with *modA2* locked variants for 3 hours. Neutrophils from two additional donors (7 & 53) were assessed following challenge with NTHi strain 723 *modA2* variants. Similar to neutrophils from donor 51 (shown in Figure 5E), challenge with *modA2* OFF caused the neutrophils to clump and become highly ruffled. In contrast, neutrophils challenged with *modA2* ON appeared more spread out and visually produced more NETs or extracellular DNA strands, compared to challenge with *modA2* OFF.

Supplementary Table 1. Rubric for scoring middle ear biofilm biomass histology.

	None (0)	Slight (1)	Moderate (2)	Significant (3)	Severe (4)
**Cell (PMN) Density**	No PMNs	< 25 %	25 % - 50 %	50 % - 75 %	> 75 %
**Strand Thickness**	No Stranding	Minimal strands; average thickness < 5 um	Most strands 5 um to 20 um, few regions of minimal stranding	Most strands 10 um -25 um with some strands > 25 um	average thickness > 25 um
